# Cortisol Levels During Acute Illnesses in Children and Adolescents: A Systematic Review

**DOI:** 10.1001/jamanetworkopen.2022.17812

**Published:** 2022-06-22

**Authors:** Mohammad Rezai, Catherine Fullwood, Beverly Hird, Meghna Chawla, Lesley Tetlow, Indraneel Banerjee, Leena Patel

**Affiliations:** 1 Brighton and Sussex University Hospitals National Health Service Trust, Brighton, United Kingdom; 2 Research & Innovation, Manchester University National Health Service Foundation Trust, Manchester, United Kingdom; 3 Centre for Biostatistics, Division of Population Health, Health Services Research & Primary Care, School of Health Sciences, University of Manchester, Manchester, United Kingdom; 4 Department of Biochemistry, Royal Manchester Children’s Hospital, Manchester, United Kingdom; 5 Department of Endocrinology, Diabetes and Metabolism, Ruby Hall Clinic Hospitals Group, Pune, Maharashtra, India; 6 Department of Paediatric Endocrinology, Royal Manchester Children’s Hospital, Manchester, United Kingdom; 7 Division of Medical Education, University of Manchester, Manchester, United Kingdom

## Abstract

**Importance:**

Endogenous cortisol levels in children and adolescents during acute illnesses can contribute to the evidence base required to optimize glucocorticoid (GC) stress doses for children and adolescents known to have GC deficiency.

**Objective:**

To identify endogenous cortisol levels during a range of acute illnesses in children and adolescents without GC deficiency from published evidence.

**Evidence Review:**

CINAHL, Cochrane Library, Cochrane Database of Systematic Reviews, Embase, and MEDLINE were searched for studies published between January 1, 2000, and June 30, 2020. Two reviewers independently identified relevant studies. Differences were resolved by joint discussion. Inclusion criteria were common acute illnesses, age from 1 month to 18 years, and basal blood cortisol levels obtained within 48 hours of presentation. Studies with fewer than 5 participants and those that included participants known to have GC deficiency or a history of treatment that could affect cortisol levels were excluded from the review. Data for predefined fields were extracted and independently checked by separate pairs of reviewers. Overall weighted means and pooled SDs for cortisol levels were calculated.

**Findings:**

All 15 studies included were hospital based and included 864 unique participants: 14 studies were prospective observational studies, 1 was part of a trial, and 5 included control individuals. Mean cortisol levels were higher in all participants with an acute illness (n = 689) than in controls (n = 175) (difference in weighted means, 18.95 μg/dL; 95% CI, 16.68-21.22 μg/dL). Cortisol levels were highest in patients with bacterial meningitis (weighted mean [pooled SD], 46.42 [22.24] μg/dL) and were more than 3-fold higher in the group with severe gastroenteritis (weighted mean [pooled SD], 39.64 [21.34] μg/dL) than in the control group. Among the subgroups with sepsis, those with shock had lower cortisol levels than those without shock (weighted mean [pooled SD], 27.83 [36.39] μg/dL vs 37.00 [23.30] μg/dL), but levels in nonsurvivors did not differ from levels in survivors (weighted mean [pooled SD], 24.89 [51.65] μg/dL vs 30.53 [30.60] μg/dL).

**Conclusions and Relevance:**

This systematic review found that, in children and adolescents without GC deficiency, circulating cortisol levels were higher during acute illnesses than those in controls and also varied across a range of acute illnesses. Whether these levels need to be achieved with exogenous GC stress doses tailored according to the nature and severity of the illness in children and adolescents with GC deficiency warrants investigation.

## Key Points

**Question:**

Do endogenous cortisol levels vary between healthy children and adolescents and those with a range of acute illnesses?

**Findings:**

In this systematic review of 15 studies including 864 unique children and adolescents with and without acute illness, mean cortisol levels varied across all acute illness groups and were higher in children and adolescents with an acute illness than in children and adolescents without an acute illness.

**Meaning:**

These findings suggest that, to achieve cortisol levels observed in children and adolescents with an intact hypothalamic-pituitary-adrenal axis during a range of acute illnesses, stress doses of glucocorticoids for children and adolescents with glucocorticoid deficiency may need to be tailored according to the nature and severity of the acute illness.

## Teaser

This systematic review of studies reporting endogenous cortisol levels in children and adolescents with and without acute illness assesses how cortisol levels vary across the different illnesses among children and adolescents without glucocorticoid deficiency.

## Introduction

Any acute physical insult to the body, including acute febrile illness, is a potential threat to well-being and survival.^1^ It triggers a physiologic stress response to maintain homeostasis. Along with complex interactions between the immune and autonomic nervous systems, the hypothalamic-pituitary adrenal axis plays a vital role in this acute stress response.^1^ The resulting rapid increase in endogenous cortisol secretion contributes to control of cardiovascular function, intravascular volume, and glucose metabolism. This inbuilt fight-or-flight response is impaired in glucocorticoid (GC) deficiency states, and a serious consequence is an acute adrenal crisis.^2^

To prevent an adrenal crisis, it is logical to hypothesize that GC replacement should mimic the normal stress-triggered physiologic increase in cortisol secretion. Standard clinical protocols for “stress doses” or “sick day doses” comprise doubling or trebling the basal GC replacement dose^3,4^ but are not based on evidence or endogenous cortisol responses from real-world scenarios in children and adolescents. Thus, further research is needed to inform appropriate GC stress dosing. The aim of this systematic review was to identify, from published evidence, endogenous cortisol levels during a range of acute illnesses in children and adolescents without GC deficiency.

## Methods

### Data Sources and Search Methods

This systematic review was approved and overseen by our hospital’s Clinical Audit Committee as a quality improvement project, and ethics committee approval was not required. A systematic review was conducted according to the Reporting Items for Systematic Reviews and Meta-analyses (PRISMA) reporting guideline.^5^ The search strategy was developed and conducted by authors with clinical expertise (I.B. and L.P.) and biochemistry expertise (B.H. and L.T.). The Healthcare Databases Advanced Search (HDAS) platform was used to search each of the following databases separately: CINAHL, Cochrane Library, Cochrane Database of Systematic Reviews, Embase, and MEDLINE.

Both keywords and thesaurus terms were used. Search terms for acute illnesses included the following: (1) *fever, febrile illness*; (2) *viral infection*, *chicken pox*, *measles*; (3) *vomiting, diarrhea, gastroenteritis*; (4) *urinary tract infection*; (5) *acute asthma*; (6) *bronchiolitis*; (7) *otitis media*; (8) *upper respiratory infection*, *common cold, sinusitis, tonsillitis, pharyngitis*; (9) *lower respiratory infection, pneumonia*; (10) *sepsis*; and (11) *critical illness*. Other keywords and medical subject headings used with Boolean operators were *plasma* OR *serum* OR *blood*; *cortisol* OR *glucocorticoid* OR *hydrocortisone* OR *adrenal cortex hormone levels*. The search terms were combined using OR, and concepts were combined using AND.

Results were limited to studies including participants aged 1 month to 18 years, published in English from January 1, 2000, to June 30, 2020, owing to lack of comparability of older cortisol assays with recent ones. For the studies found, manual searches of their reference lists were carried out; searches for other eligible studies that cited them and further work by their authors were also conducted.

### Study Selection, Screening, and Eligibility Criteria

All search results were saved in electronic files, and abstracts were accessed online. Titles and abstracts were screened for eligibility criteria. A PRISMA flow diagram is shown in the eFigure in the Supplement.

The inclusion criteria for the studies were (1) acute illnesses commonly encountered during childhood and adolescence; (2) participants aged 1 month to 18 years; (3) basal plasma or serum cortisol levels in venous or arterial blood (but not after stimulation with synthetic corticotropin, corticotrophin-releasing hormone, or metyrapone), measured within 48 hours of presentation (irrespective of time of day because the diurnal pattern of cortisol secretion is lost during acute illness); and (4) blood cortisol values reported in the results. Studies that met these criteria were included independent of the primary aim of the study.

The following exclusion criteria were applied: (1) case reports or series of fewer than 5 participants; (2) participants younger than 1 month or older than 18 years; (3) participants known to have GC deficiency, adrenal insufficiency, or adrenal deficiency; (4) treatment with a systemic GC before the acute illness; (5) GC treatment given before blood was taken for measurement of cortisol levels; (6) other treatment with potential influence on cortisol levels (eg, etomidate); (7) cortisol or its metabolites measured in saliva or urine; and (8) blood cortisol metabolites (eg, 17-hydroxycorticosteroid) measured. Two reviewers (M.R. and M.C.) independently read the abstracts and used these criteria to screen for study eligibility. Thereafter, the full text of each study was read to classify it as relevant or not relevant. Differences were resolved by joint review and discussion of the study.

### Data Extraction

From relevant studies, data for the following predefined fields were extracted by 2 of us (M.R. and M.C.) and checked by 2 others (I.B. and L.P.) independently: country, study design, number of participants, age, sex, timing of cortisol sample, cortisol assay, cortisol levels (mean, median, SD, SEM, IQR, 95% CI, or range), and reference range. For patients, data were also extracted about prior health, prior GC treatment, and nature and severity of the acute illness. The outcome of primary interest was the blood cortisol level and spread for control participants and patients in different acute illness groups.

When data could not be ascertained for a study, the final outcome reflects the mean and SD across the remaining studies. Because data were only missing for the descriptive age variable, rather than the primary outcome cortisol level, it was not deemed necessary or appropriate to use any imputation or sensitivity methods.

### Statistical Analysis

For the purpose of the study, cortisol values presented in SI units (nanomoles per liter) were converted using the factor 1/27.6 into conventional units (micrograms per deciliter). All cortisol statistics were converted to mean and SD according to assumptions made by Hozo et al^6^ and Wan et al.^7^ Overall means and SDs were calculated using means weighted by study sample size under a fixed-effects model and pooled SDs. To compare groups, the difference of the weighted means and corresponding 95% CIs are presented. All analyses were performed using R, version 3.6.0 (R Foundation for Statistical Computing)^8^ and the R package meta.^9^

## Results

### Characteristics of Included Studies

Of 345 records screened and assessed, 15 studies including 864 unique participants were included (eFigure and eTable in the Supplement).^10,11,12,13,14,15,16,17,18,19,20,21,22,23,24^ All studies were hospital based: 14 were prospective observational studies, and 1 was part of a trial.^13^ From 1 study,^21^ the subgroup treated with etomidate was excluded, but other subgroups were included. Five studies had controls (n = 175): healthy children and adolescents in 1 study,^23^ pediatric patients before elective minor surgery in 3 studies,^17,22,24^ and patients after recovery from the acute illness in 2 studies.^15,17^ Some studies reported the number of girls and boys among the participants but not differences in cortisol values by sex. However, none reported pubertal status.

No studies were found for fever or febrile illness, viral infection, vomiting, urinary tract infection, otitis media, and upper or lower respiratory infection. Patients (n = 689) were grouped by the acute illness and its severity as shown in the Table. Except for sepsis (n = 10), the numbers of studies for each group were small (1-3 studies). The subgroup sample sizes within the studies ranged from 7 to 89.

**Table.** Blood Cortisol Levels for Control Participants and Different Acute Illness Groups

Participant groups and acute illness subgroups	No. of studies	No. of participants	Weighted mean (pooled SD)		Cortisol comparison	Difference between weighted means (95% CI), μg/dL
			Age, y	Cortisol level, μg/dL		
Control participants	5	175	2.28 (1.97)^a^	10.44 (5.86)	NA	NA
Bronchiolitis, all	3	123	0.37 (0.20)	19.09 (13.00)	Bronchiolitis vs controls	8.65 (6.20 to 11.11)
Bronchiolitis, mild or moderate	3	60	0.42 (0.17)	16.14 (10.12)	Severe vs mild or moderate bronchiolitis	5.75 (1.20 to 10.30)
Bronchiolitis, severe	3	63	0.33 (0.22)	21.89 (15.23)		
Gastroenteritis, severe	1	52	3.20 (3.64)	39.64 (21.34)	Gastroenteritis vs controls	29.20 (23.34 to 35.07)
Meningitis, all	2	40	4.61 (6.74)	41.34 (20.24)	Meningitis vs controls	30.90 (24.57 to 37.23)
Aseptic meningitis	1	14	6.60 (10.64)	31.90 (15.90)	Bacterial meningitis vs aseptic	14.52 (2.59 to 26.46)
Bacterial meningitis	2	26	3.54 (2.95)	46.42 (22.24)		
Sepsis, all	10	430	5.22 (8.44)	30.58 (32.98)	Sepsis vs controls	20.15 (16.91 to 23.38)
Sepsis, no shock	4	129	4.34 (5.49)	37.00 (23.30)	Sepsis, no shock vs shock	9.17 (3.42 to 14.93)
Sepsis, shock	9	301	5.65 (9.68)^b^	27.83 (36.39)		
Sepsis, survived	5	150	5.63 (11.35)^c^	30.53 (30.60)	Sepsis, survived vs not survived	5.65 (−10.72 to 22.02)
Sepsis, did not survive	4	42	2.05 (2.83)^d^	24.89 (51.65)		
Critical illness other than sepsis	2	44	7.34 (5.34)	23.53 (18.82)	Critical illness other than sepsis vs controls	13.10 (7.47 to 18.73)
All illness groups	15	689	4.25 (6.92)^b^	29.39 (28.12)	All illness groups vs controls	18.95 (16.68 to 21.22)

Abbreviation: NA, not applicable.

SI conversion factor: To convert cortisol level to nmol/L, multiply by 27.6.

^a^ One study missing for mean and 4 studies for SD.

^b^ Three studies missing for mean and 5 studies for SD.

^c^ One study missing for mean and 2 studies for SD.

^d^ Two studies missing for mean and 3 studies for SD.

For all 5 studies with controls, blood for cortisol measurement was drawn between 8 and 9 am.^15,17,22,23,24^ Among patients with acute illnesses, samples were taken between 8 and 9 am and within 24 hours of admission in 3 studies,^17,19,22^ within 48 hours of admission in 1 study,^15^ and at the time of admission in 11 studies.^10,11,12,13,14,16,18,20,21,23,24^

Cortisol measurement was primarily performed by nonisotopic immunoassay: competitive chemiluminescence in 7 studies^10,11,13,19,20,23,24^ and enzyme-linked immunosorbent assay in 2 studies.^15,18^ Radioimmunoassay was used in 3 studies,^14,17,22^ and the method was not stated in 3 studies.^12,16,21^

### Cortisol Levels During Acute Illness

Compared with controls (weighted mean [pooled SD] 10.44 [5.86] μg/dL), cortisol levels were higher in all groups (29.39 [28.12] μg/dL) (difference between weighted means, 18.95 μg/dL; 95% CI, 16.68-21.22 μg/dL) (Table). Cortisol levels were highest in patients with bacterial meningitis^11,18^ and more than 4-fold higher than those in controls (weighted mean [pooled SD], 46.42 [22.24] μg/dL vs 10.44 [5.86] μg/dL). Cortisol levels were lowest in patients with mild or moderate bronchiolitis^15,17,22^ (weighted mean [pooled SD], 16.14 [10.12] μg/dL). These findings are presented in the Figure.

**Figure.**
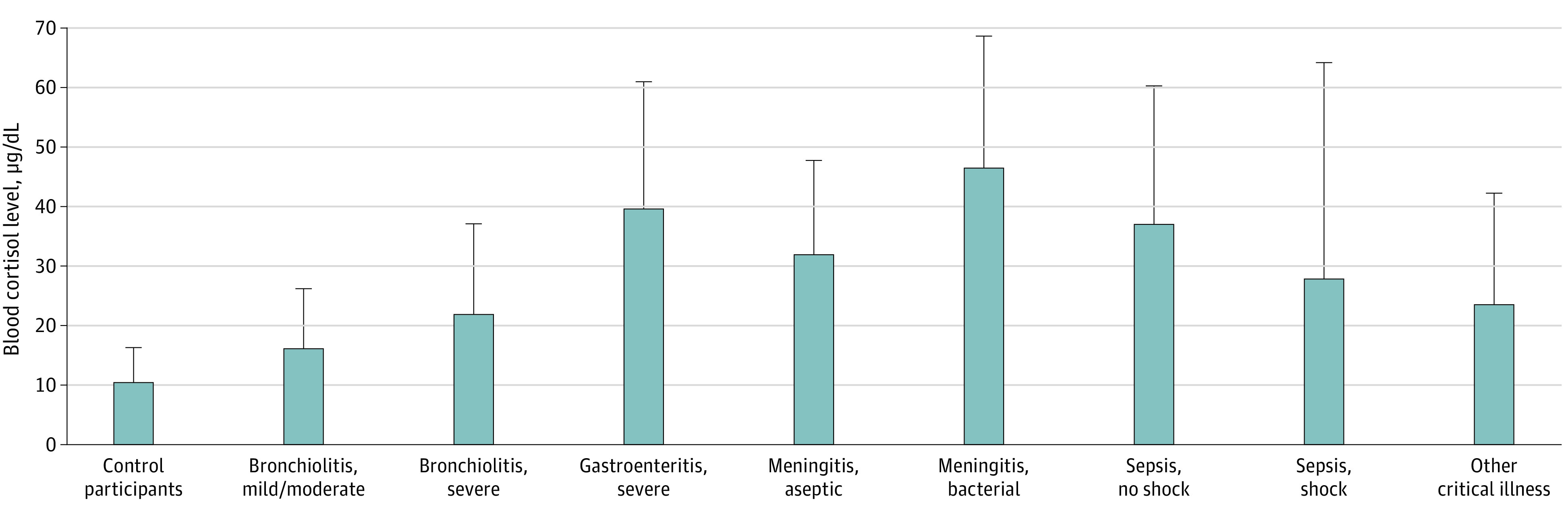
Weighted Means and Pooled SDs for Cortisol Levels From 15 Studies Reviewed Error bars indicate SDs. To convert cortisol levels to nmol/L, multiply by 27.6.

Among the subgroups with sepsis, those with shock^10,11,13,14,19,20,21,23,24^ had lower cortisol levels than those without shock^12,13,21,24^ (difference, 9.17 μg/dL; 95% CI, 3.42-14.93 μg/dL), but levels in survivors^10,13,14,19,20^ did not differ from levels in nonsurvivors^10,13,19,20^ (difference, 5.65 μg/dL; 95% CI, −10.72 to 22.02 μg/dL) (Figure).

Cortisol levels were higher in patients with severe gastroenteritis requiring intravenous fluid rehydration (mean [SD], 39.64 [19.09] μg/dL)^16^ compared with patients with bronchiolitis^15,17,22^ (weighted mean [pooled SD], 19.09 [13.00] μg/dL; difference between weighted means, 20.55 μg/dL; 95% CI, 14.31-26.79 ug/L), sepsis (weighted mean [pooled SD], 30.58 [32.98] μg/dL; difference, 9.06 μg/dL; 95% CI, 2.47-15.64 μg/dL),^10,11,12,13,14,19,20,21,23,24^ or other critical illness (difference, 16.10 μg/dL; 95% CI, 8.07-24.14 μg/dL)^23,24^ but were not significantly different from levels in patients with aseptic meningitis (mean [SD], 31.9 [15.9] μg/dL; difference, 7.74 μg/dL; 95% CI, −2.41 to 17.89 μg/dL).^18^ Cortisol levels in the combined subgroups with noncritical illnesses (weighted mean [pooled SD], 28.20 [16.84] μg/dL) did not differ significantly from those in patients with critical illness including sepsis (weighted mean, [pooled SD] 29.61 [31.35] μg/dL) (difference, 1.41 μg/dL; 95% CI, −2.15 to 4.97 μg/dL).

## Discussion

According to a recent study by Worth et al,^25^ the rate of death due to an adrenal crisis among children with adrenal insufficiency has remained high. The correct GC stress dose for different acute illnesses has the potential to prevent death.^23^ Glucocorticoid stress doses for children and adolescents have been based on opinion owing to a dearth of evidence. To contribute toward closing this gap in evidence, we conducted this systematic review of published blood cortisol levels at the time of acute illness in children and adolescents without GC deficiency, using newer assays available in the past 2 decades. The basal cortisol levels between 8 and 9 am in controls are consistent with those reported by Eyal et al^26^ (mean [SD], 11.53 [5.74] μg/dL). Comparing these with cortisol levels observed in children and adolescents during a range of acute illnesses that required hospitalization suggests that GC stress doses may need to be tailored according to the nature and severity of the illness in children and adolescents with GC deficiency and warrant further research.

Cortisol levels did not differ significantly between the combined subgroups with noncritical illnesses and the groups with critical illness including sepsis. However, the paradoxically higher cortisol levels associated with gastroenteritis requiring intravenous fluids in critically ill children and adolescents suggest an inappropriate cortisol response to acute stress. In addition, the cause of sepsis, such as meningococcal disease, which is known to be associated with adrenal hemorrhage, could impair the true GC stress response. Sepsis has been extensively investigated with increasing evidence of maladaptive homeostasis, functional GC deficiency, and higher mortality in those with very high or very low cortisol levels.^27^ From studies in adults and mice, greater understanding has emerged for the corticotropin-cortisol dissociation in critical illness.^28,29,30,31^ Téblick et al^31^ found high circulating cortisol levels to be associated with central activation of the hypothalamus and increased pro-opiomelanocortin expression in the pituitary. However, corticotropin levels were low, reflecting likely impaired cleavage from pro-opiomelanocortin. In turn, increased pro-opiomelanocortin secreted by the pituitary was hypothesized to directly stimulate adrenal secretion of cortisol.^31^ Such experiments in GC deficiency states will shed light on our understanding of the complex pathophysiologic adaptations in the hypothalamic-pituitary-adrenal axis and peripheral tissues during critical illness, with and without stress doses of GC, and will provide insight into optimizing GC replacement. In addition, elegant studies of critically ill adults have shown that GC replacement must be administered as a continuous infusion of hydrocortisone, and further studies are required in children and adolescents.^32^ For the day-to-day management of GC deficiency and novel methods of delivering GC replacement that are more physiologic than currently available treatments, we refer the reader to reviews by Hindmarsh^33^ and Porter et al.^34^

### Limitations

This study has limitations. One limitation is that all eligible studies were hospital based. Thus, there was a paucity of publications for many common illnesses in children and adolescents, including those typically managed in the community. We found no recent studies on upper respiratory and urinary tract infections and other common childhood illnesses. In a historical report,^35^ mean (SD) cortisol levels, measured by enzyme-linked immunoassay, ranged from 8.30 (3.80) μg/dL during fever of unknown origin to 11.81 (10.72) μg/dL during pneumonia. In addition, the control groups in the studies we reviewed were relatively small, and only 1 of the 5 studies with controls included healthy children and adolescents; in the other studies, children and adolescents who were scheduled for but had not undergone elective minor surgery and children and adolescents who had recovered from the acute illness served as controls.

Another limitation is the crude comparisons for cortisol levels because there are no validated methods to adjust for interassay differences.^36^ In addition, these differences may be accentuated in acute illness owing to a decrease in cortisol-binding globulin concentrations and an increase in circulating free cortisol.^37^ Because older assays may not compare well with current methods, we excluded studies completed before 2000. Despite restricting our review to publications in the past 2 decades, 3 studies in our review used older radioimmunoassay technology, and 3 did not state the assay used.

A third limitation is the variety of methods used to report cortisol levels. To convert values reported as median, IQRs, or minimums and maximums to means and SDs, assumptions had to be made in the absence of raw data.

## Conclusions

In this systematic review of studies on children and adolescents with an intact hypothalamic-pituitary-adrenal axis, circulating cortisol levels during acute illness were found to be higher than in controls and varied among a range of acute illnesses. Robust studies are required to examine whether comparable levels need to be achieved with exogenous GC treatment when children and adolescents with GC deficiency experience different acute illnesses.

## Notes

Supplement. **eFigure.** PRISMA Flow Chart for Search Record **eTable.** Details of the 15 Studies Included in the Systematic Review
