# Supplementary material for: Cortisol Levels During Acute Illnesses in Children and Adolescents: A Systematic Review
Source: JAMA Netw Open. 2022 Jun 22;5(6):e2217812. doi: 10.1001/jamanetworkopen.2022.17812 (PMC9218852; doi:10.1001/jamanetworkopen.2022.17812)
Supplement: Supplement. — eFigure. PRISMA Flow Chart for Search Record eTable. Details of the 15 Studies Included in the Systematic Review [file jamanetwopen-e2217812-s001.pdf]

Supplementary Online Content

Rezai M, Fullwood C, Hird B, et al. Cortisol levels during acute illnesses in children and adolescents: a systematic review. *JAMA Netw Open*. 2022;5(6): e2217812. doi:10.1001/jamanetworkopen.2022.17812

eFigure. PRISMA Flow Chart for Search Record

eTable. Details of the 15 Studies Included in the Systematic Review

This supplementary material has been provided by the authors to give readers additional information about their work.

© 2022 Rezai M et al. *JAMA Network Open*.

eFigure. PRISMA flow chart for Search record

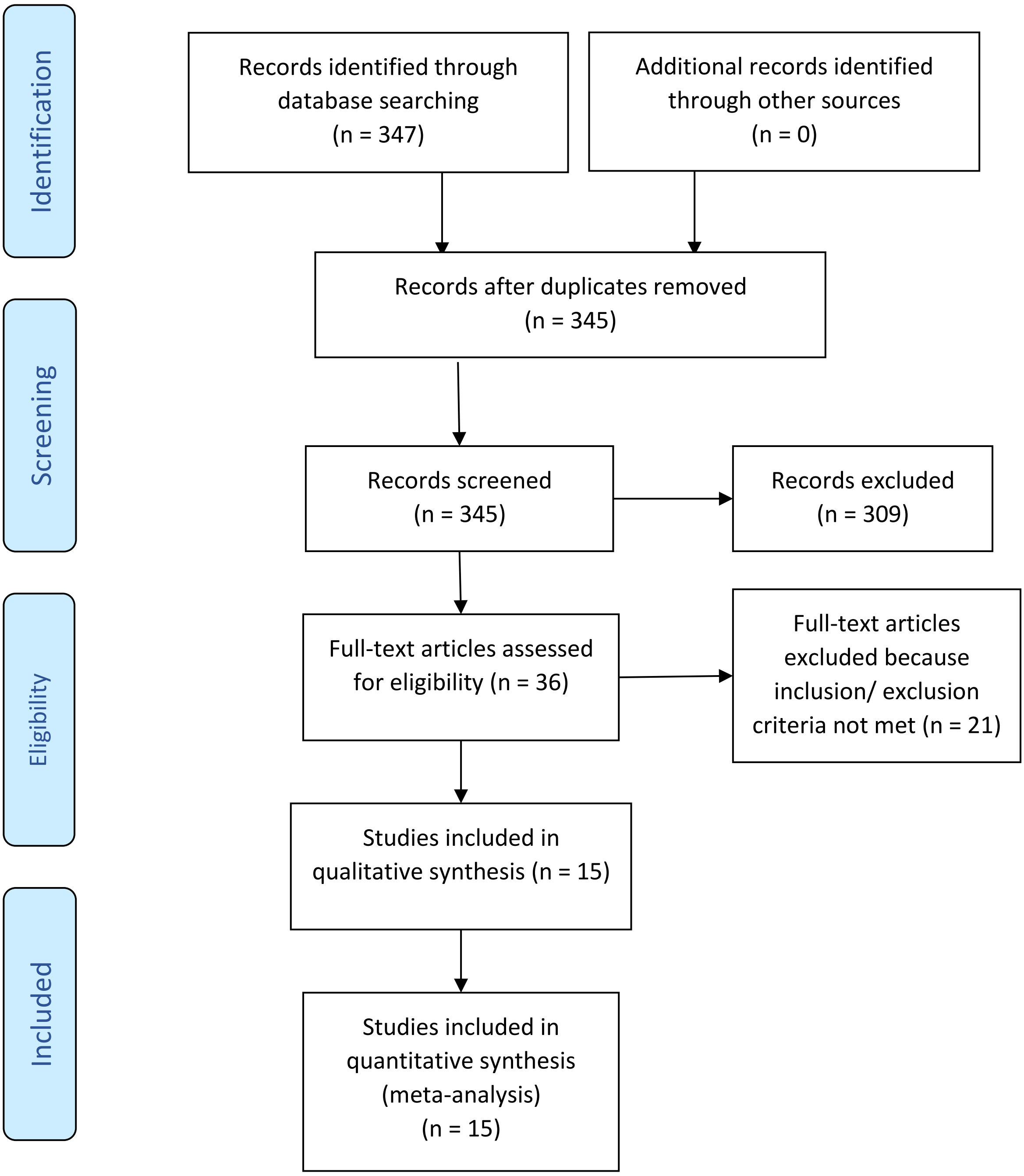

© 2022 Rezai M et al. *JAMA Network Open*.

eTable 1. Details of the 15 studies included in the systematic review

Author (Year)	Country	Study type	Acute illness	Population size
Joosten (2000) [10]	Netherlands	Prospective observational, 2 groups	Meningococcal septic shock survived, nonsurvivors	26
van Woensel (2001) [11]	Netherlands	Prospective observational, 3 subgroups	Meningococcal meningitis without and with sepsis, and fulminant sepsis	32
Bone (2002) [12]	UK	Prospective, single group	Meningococcal disease	65
de Kleijn (2002) [13]	Netherlands	Prospective, part of a trial, 3 subgroups	Meningococcal sepsis with no shock, shock and survived, nonsurvivors	62
Lichtarowicz-Krynska (2004) [14]	UK	Prospective, single group	Meningococcal disease	31
Tasker (2004) [15]	UK	Prospective controlled observational, two subgroups during acute illness and at recovery	Moderate and severe bronchiolitis	32
Neville (2005) [16]	Australia	Prospective observational, single group	Acute gastroenteritis requiring intravenous fluid rehydration	52
Pinto (2006) [17]	Chile	Prospective controlled observational, two subgroups and at recovery; controls before elective minor surgery	Mild and severe bronchiolitis	42
Singhi (2006) [18]	India	Prospective observational, 2 groups	Aseptic meningitis; bacterial meningitis	30
Casartelli (2007) [19]	Brazil	Prospective observational, 2 subgroups	Septic shock survived and nonsurvivors	22
Sarthi (2007) [20]	India	Prospective, single group	Septic shock	30
den Brinker (2008) [21]	Netherlands	Prospective observational, 2 subgroups	Meningococcal sepsis not intubated, intubated without etomidate	37

© 2022 Rezai M et al. *JAMA Network Open*.

Diaz (2012) [22]	Chile	Prospective controlled observational, two subgroups and controls before elective minor surgery	Mild/moderate and severe bronchiolitis	49
Karaguzel (2012) [23]	Turkey	Prospective controlled observational, 2 groups; age matched healthy controls	Severe sepsis; acute critical illness without sepsis	50
Alder (2018) [24]	USA	Prospective controlled observational, 3 subgroups and controls before elective hernia surgery	Sepsis and septic shock; Systemic inflammatory response syndrome	129

© 2022 Rezai M et al. *JAMA Network Open*.
